# Drivers and Performance Outcomes of Social Responsibility Accounting: An Integrated Model for SMEs in the Mekong Delta

**DOI:** 10.12688/f1000research.179819.1

**Published:** 2026-06-25

**Authors:** Phong Nguyen Thien, Hang Tran Thuy, Hien Dinh Cong

**Affiliations:** 1 Department of Accounting - Finance - Banking, Tay Do university, Can Tho City, 94000, Vietnam

**Keywords:** Social Responsibility Accounting, Corporate Social Responsibility, SMEs, Firm Performance, Stakeholder Theory, Emerging Economy, Vietnam

## Abstract

**Background:**

While the strategic importance of Social Responsibility Accounting (SRA) is well-established for large corporations, its antecedents and firm-level outcomes in the context of Small and Medium-sized Enterprises (SMEs) in emerging economies remain critically under-explored. This study develops and empirically tests an integrated theoretical model examining the key drivers of SRA implementation and its subsequent impact on firm performance and access to finance among SMEs in Vietnam.

**Methods:**

Employing a sequential exploratory mixed-method design, qualitative interviews with nine experts informed the development of a survey instrument, which was then used to collect quantitative data from 362 SMEs in the Mekong Delta region. The proposed model was analyzed using Partial Least Squares Structural Equation Modeling (PLS-SEM).

Results: The results confirm that stakeholder pressure, leadership commitment, accounting department competence, and export orientation are significant positive drivers of SRA implementation. In turn, SRA implementation was found to be a strong and significant predictor of both enhanced firm performance and improved access to finance.

**Conclusions:**

This study provides a comprehensive empirical examination of the SRA ecosystem within SMEs in an emerging market. It offers a nuanced understanding of why SMEs adopt SRA and confirms its strategic role as a mediating mechanism in improving both operational and financial outcomes.

## 1. Introduction

In the context of Vietnam’s increasing global economic integration, sustainable development has emerged as a pivotal strategic issue for national competitiveness ( T. H. T. Nguyen & Hens, 2015; World Bank, 2021). Consequently, Social Responsibility Accounting (SRA)—a formal process for measuring, disclosing, and being accountable for organizational performance towards society and the environment—has transitioned from a niche academic topic to a corporate imperative ( Bebbington et al., 2017; Gray et al., 1996). While research on SRA in Vietnam has gained traction, studies have predominantly focused on large, listed corporations, often driven by mandatory disclosure requirements ( Hoang et al., 2017; T. H. T. Nguyen & Hens, 2015). A significant research gap persists regarding SRA adoption within Small and Medium-sized Enterprises (SMEs), which constitute the backbone of the economy yet operate under vastly different constraints and pressures ( Lepoutre & Heene, 2006; Spence, 2007). This gap is particularly critical in regions of combined economic and ecological importance, such as the Mekong Delta.

The Mekong Delta, Vietnam’s agricultural and aquacultural heartland, represents a unique confluence of immense economic opportunity and acute environmental vulnerability. As a key node in global food supply chains, the region’s SMEs are vital to the national economy ( General Statistics Office of Vietnam, 2022). However, this region is also on the frontline of climate change, facing existential threats from sea-level rise, salinity intrusion, and ecological degradation ( IPCC, 2022; Neumann et al., 2015). For SMEs operating here, the pressures to adopt sustainable practices are twofold: normative pressures from international buyers demanding transparent and responsible sourcing ( Gereffi et al., 2005), and coercive pressures from a fragile ecosystem where the consequences of unsustainable operations are immediate and severe. Despite their collective impact, these resource-constrained firms face significant challenges in adopting non-traditional practices like SRA ( Murillo & Lozano, 2006), making it crucial to understand the factors that can facilitate this transition.

Therefore, this study aims to address this critical gap by investigating the key factors influencing the implementation of SRA among SMEs in the Mekong Delta region. Specifically, this research seeks to: (1) identify the primary external and internal factors affecting SRA implementation; (2) develop and test a model that explains the interrelationships between these drivers and SRA implementation; and (3) examine the subsequent impact of SRA implementation on key organizational outcomes, such as firm performance and access to finance. The findings are expected to offer significant theoretical contributions by validating an SRA model for SMEs in an emerging market context characterized by high environmental vulnerability. Practically, this study will provide actionable insights for SME managers and policymakers seeking to foster sustainable development within one of Southeast Asia’s most vital economic regions.

## 2. Literature review and hypotheses

### 2.1 Theoretical framework

This study develops an integrated theoretical framework by synthesizing several complementary theories to explain the antecedents and consequences of Social Responsibility Accounting (SRA) implementation in the specific context of Small and Medium-sized Enterprises (SMEs). We argue that SRA implementation is a complex phenomenon driven by a confluence of external pressures, internal capabilities, and strategic choices, which in turn generates tangible organizational outcomes. Our framework primarily draws upon Institutional Theory, Stakeholder Theory, the Resource-Based View (RBV), Upper Echelons Theory, and Signaling Theory.

The adoption of SRA can be first understood through the external pressures exerted on the firm. Institutional Theory posits that organizations conform to the norms, rules, and beliefs of their wider environment to gain legitimacy and ensure survival ( DiMaggio & Powell, 1983; Meyer & Rowan, 1977). These institutional pressures are often transmitted through key stakeholders. Complementing this, Stakeholder Theory argues that firms must attend to the interests of a wide range of constituents beyond just shareholders to achieve long-term success ( Freeman, 1984; Donaldson & Preston, 1995). Together, these theories suggest that SMEs face coercive pressures from regulators and normative pressures from customers and communities, compelling them to adopt practices like SRA to demonstrate social legitimacy ( Deegan, 2002). Furthermore, for firms engaged in global commerce, the institutional environment extends to international markets, where pressures for environmental and social compliance are often more stringent ( Christmann & Taylor, 2001). This theoretical lens provides the foundation for our hypotheses regarding Stakeholder Pressure (H1) and Export Orientation (H4).

While external pressures create the impetus for change, a firm’s internal context determines its capacity and willingness to respond. The Resource-Based View (RBV) contends that a firm’s competitive advantage stems from its unique, valuable, and inimitable internal resources and capabilities ( Barney, 1991; Wernerfelt, 1984). In the context of SRA, a highly competent accounting department represents a critical human capital resource, enabling the firm to navigate the complexities of non-financial measurement and reporting ( Schaltegger & Burritt, 2010). This perspective underpins our hypothesis on Accounting Department Competence (H3). However, the deployment of such resources is not automatic; it is a matter of strategic choice. Here, Upper Echelons Theory becomes critical, asserting that organizational outcomes are a reflection of the values and cognitions of its top managers ( Hambrick & Mason, 1984; Hambrick, 2007). This is particularly salient in SMEs, where the owner-manager’s vision is paramount ( Spence, 2007). A strong leadership commitment to sustainability will thus direct firm resources towards SRA implementation, providing the rationale for our hypothesis on Leadership Commitment (H2).

Finally, our framework extends to the consequences of SRA implementation, arguing that it is not merely a cost but a strategic, value-creating activity. The integration of RBV and Stakeholder Theory suggests that by implementing SRA, firms can build valuable intangible assets such as reputation and trust, leading to enhanced Firm Performance (H5) ( Orlitzky et al., 2003; Hart & Dowell, 2011). Furthermore, Signaling Theory posits that firms can reduce information asymmetry by sending credible signals about their underlying quality to external parties ( Spence, 1973; Connelly et al., 2011). Transparent SRA reporting acts as such a signal to financial institutions, indicating robust non-financial risk management and a long-term orientation. This reduces perceived risk for lenders and investors, thereby improving the firm’s Access to Finance (H6) ( Cheng et al., 2014).

In summary, this multi-theoretic framework provides a holistic view, explaining SRA implementation as a function of external institutional pressures and internal strategic and operational capabilities, which in turn influences critical performance and financial outcomes.

### 2.2 Hypotheses development

This section integrates foundational theories to propose a comprehensive model that explains both the antecedents and outcomes of Social Responsibility Accounting (SRA) implementation in Small and Medium-sized Enterprises (SMEs).

**2.2.1 Stakeholder pressure**

Grounded in Stakeholder Theory, a firm’s long-term success is contingent upon its ability to manage relationships with a wide array of constituents, not just shareholders ( Freeman, 1984; Donaldson & Preston, 1995). These stakeholders, including customers, regulators, and the community, exert significant institutional pressures that compel firms to adopt socially responsible behaviors to maintain social legitimacy ( Deegan, 2002; Scott, 2005). SRA implementation serves as a formal mechanism for firms to respond to these demands for transparency and accountability ( Gray et al., 1996). Empirical studies have consistently shown that pressure from primary stakeholders is a powerful driver of corporate environmental and social strategies (e.g., Henriques & Sadorsky, 1999; Buysse & Verbeke, 2003). Therefore, we posit:

H1:
*Stakeholder pressure positively influences the implementation of Social Responsibility Accounting in SMEs.*

**2.2.2 Leadership commitment**

Upper Echelons Theory posits that organizational strategies are a reflection of top management’s values and cognitive frameworks ( Hambrick & Mason, 1984; Hambrick, 2007). In the SME context, where the owner-manager’s influence is often absolute, their personal commitment to ethical values and sustainable strategies is the primary catalyst for resource allocation to discretionary activities like SRA ( Spence, 2007). Leaders who perceive sustainability as a core value are more likely to champion the necessary organizational changes and foster a supportive culture, transforming intention into concrete action ( Aragón-Correa et al., 2008). Thus:

H2:
*Leadership commitment positively influences the implementation of Social Responsibility Accounting in SMEs.*

**2.2.3 Accounting department competence**

The Resource-Based View (RBV) suggests that a firm’s competitive advantage stems from its unique, valuable, and inimitable internal capabilities ( Barney, 1991; Wernerfelt, 1984). SRA is not a simple task; it requires specialized skills to identify, measure, and report complex non-financial information, a capability that extends beyond traditional accounting ( Schaltegger & Burritt, 2010). A competent accounting department, therefore, represents a critical organizational capability that enables the firm to implement SRA effectively and credibly. Without this specific human capital, firms face a significant internal barrier to translating their sustainability ambitions into systematic practice ( Latan et al., 2018). Accordingly:

H3:
*The competence of the accounting department positively influences the implementation of Social Responsibility Accounting in SMEs.*

**2.2.4 Export orientation**

From an Institutional Theory perspective, firms operating in international markets face intense coercive and normative pressures to conform to global standards ( DiMaggio & Powell, 1983). Major import markets, particularly in Europe and North America, increasingly demand that suppliers in global value chains demonstrate adherence to stringent environmental and labor standards ( Christmann & Taylor, 2001; Gereffi et al., 2005). For SMEs, export orientation thus acts as a powerful institutional force, incentivizing the adoption of SRA as a strategic tool to signal compliance, mitigate risks, and maintain access to lucrative international markets. Hence, we hypothesize:

H4:
*Export orientation positively influences the implementation of Social Responsibility Accounting in SMEs.*

**2.2.5 Firm performance**

The relationship between corporate social responsibility and financial performance has been a central theme in management literature. An integrated perspective of Stakeholder Theory and the RBV suggests that SRA is not merely a cost but a strategic investment that builds valuable intangible assets like reputation and stakeholder trust ( Hillman & Keim, 2001). By proactively managing stakeholder relationships and improving resource efficiency, firms can enhance their competitive advantage. This argument is particularly relevant for SMEs, where subjective assessments of organizational performance are often used due to the limited availability of objective financial data ( Dess & Robinson, 1984) A large body of meta-analytic evidence confirms a positive, albeit complex, relationship between corporate social performance and financial performance ( Orlitzky et al., 2003; Margolis et al., 2009). Therefore, we expect:

H5:
*The implementation of Social Responsibility Accounting positively influences firm performance.*

**2.2.6 Access to finance**

Signaling Theory posits that firms can reduce information asymmetry by sending credible signals about their quality and long-term viability to external capital providers ( Spence, 1973; Connelly et al., 2011). Transparent SRA reporting acts as a powerful, positive signal to financial institutions, indicating that the firm has robust systems for managing non-financial (e.g., environmental, social, and governance-related) risks ( Dhaliwal et al., 2011). This enhanced transparency and perceived lower risk can reduce the cost of capital and improve a firm’s ability to secure loans and investments, a benefit particularly crucial for capital-constrained SMEs ( Cheng et al., 2014). Thus, we propose:

H6:
*The implementation of Social Responsibility Accounting positively influences SMEs’ access to finance.*

**Figure 1.**
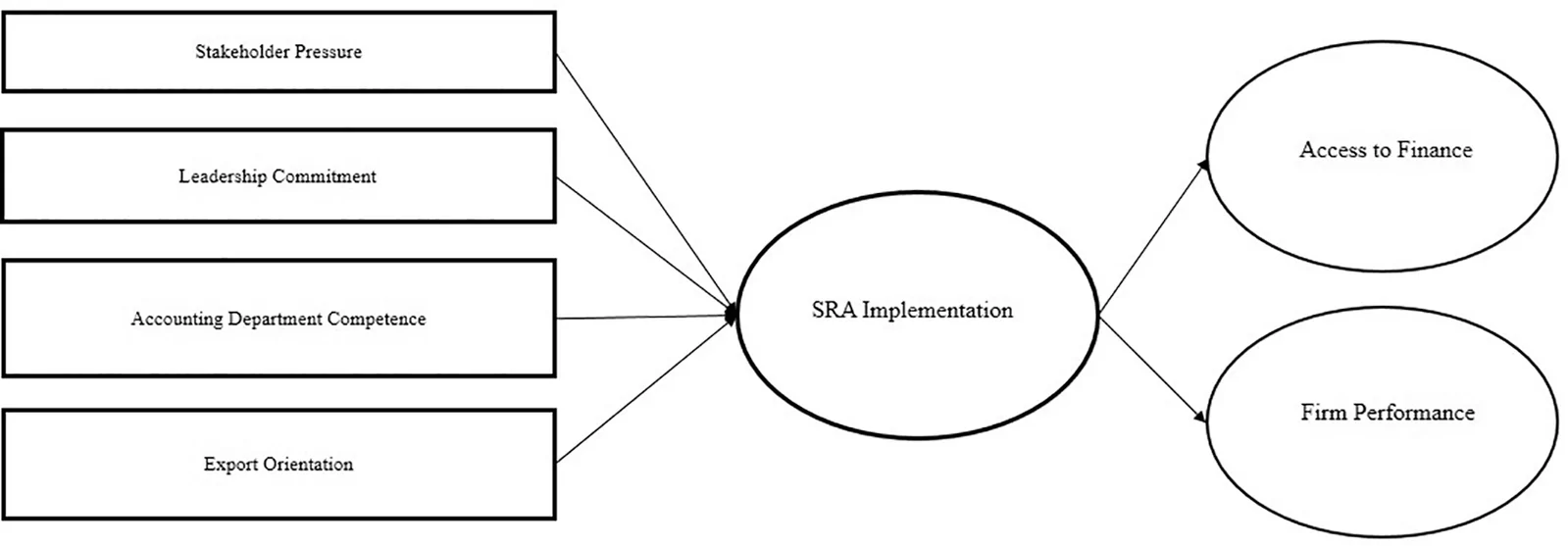
Official research model. Source: Proposed by the Author.

The proposed relationships are illustrated in Figure 1.

## 3. Research methodology

### 3.1 Research design

This study employed a sequential exploratory mixed-method design ( Creswell & Plano Clark, 2018). An initial qualitative phase was conducted to explore and validate the research constructs within the local context, which subsequently informed the development and refinement of the instrument for the main quantitative phase. This approach ensures that the measurement scales are not only theoretically grounded but also practically relevant to the target population.

### 3.2 Measurement instrument and data collection

The initial qualitative phase consisted of in-depth interviews with a purposively selected panel of nine experts. This panel included academics, senior managers, and chief accountants with extensive experience within the SME sector of the Mekong Delta. The primary objective of these interviews was to validate the relevance of the adapted constructs and to refine the clarity and wording of the survey items for the regional context.

Following this qualitative refinement, the main quantitative phase was conducted using a cross-sectional survey. The target population was Small and Medium-sized Enterprises (SMEs) operating across key provinces of the Mekong Delta region. To ensure representativeness of the region’s diverse economic activities (e.g., agriculture, aquaculture, manufacturing, and services), the sample was drawn from firms located in several key provinces, including Can Tho, An Giang, and Kien Giang. A structured questionnaire was developed using measurement scales adapted from established literature (e.g., Freeman, 1984; Carroll, 1991; Alkisher, 2013). All constructs were measured on a five-point Likert scale, ranging from 1 (“Strongly Disagree”) to 5 (“Strongly Agree”). The finalized instrument was distributed to 400 SME managers selected across these provinces. A total of 362 valid responses were collected and deemed usable for analysis, resulting in a high response rate of 90.5%.

Informed consent was obtained from all individual participants included in the study. Before participating in the survey, respondents were informed about the study’s purpose, the voluntary nature of their participation, and the strict confidentiality of their responses. Written consent was deemed unnecessary and verbal consent was obtained instead to ensure complete anonymity of the respondents, which is crucial when collecting data from SMEs regarding internal organizational practices.

### 3.3 Data analysis method

The dataset was subsequently analyzed using Partial Least Squares Structural Equation Modeling (PLS-SEM) with the SmartPLS 4 software ( Ringle et al., 2024). PLS-SEM was selected over Covariance-Based SEM (CB-SEM) for its distinct advantages in this research context. Specifically, it is well-suited for prediction-oriented models, handles complex models with numerous constructs and indicators effectively, and does not require stringent assumptions regarding normal data distribution ( Hair et al., 2019; Henseler et al., 2009).

Following the recommendations of Hair et al. (2022), a two-step analytical procedure was employed: assessment of the measurement model, followed by the assessment of the structural model.

**3.3.1 Measurement model assessment**

The measurement model was evaluated to ensure its reliability and validity. First, internal consistency reliability was assessed using Cronbach’s Alpha and Composite Reliability (CR). A value of 0.70 or higher for both indicators is considered acceptable ( Hair et al., 2019). Second, convergent validity was established by examining outer loadings and the Average Variance Extracted (AVE). Outer loadings should be above 0.70, and the AVE for each construct should exceed the 0.50 threshold ( Hair et al., 2022). Third, discriminant validity was assessed using the Heterotrait-Monotrait (HTMT) ratio of correlations, with values below the conservative threshold of 0.85 confirming that each construct is empirically distinct ( Henseler et al., 2015).

**3.3.2 Structural model assessment**

Once the measurement model was confirmed to be robust, the structural model was evaluated. First, collinearity diagnostics were performed by examining the Variance Inflation Factor (VIF) for all predictor constructs; values below 5 indicated that multicollinearity was not a concern ( Hair et al., 2019). Next, to test the research hypotheses, a bootstrapping procedure with 5,000 resamples was executed to determine the statistical significance of the path coefficients (β) ( Hair et al., 2022). The model’s explanatory power was assessed via the coefficient of determination (R ^2^), while the substantive impact of each predictor was evaluated using the effect size (f ^2^). Finally, the model’s out-of-sample predictive relevance was determined using the Stone-Geisser’s Q ^2^ value, where a value greater than zero indicates predictive relevance ( Hair et al., 2019).

## 4. Research findings

### 4.1 Sample characteristics

The demographic and firm ographic characteristics of the final sample (N = 362) are summarized in Table 1. The sample is geographically distributed across the Mekong Delta, with a significant concentration in Can Tho City (40.1%), the region’s economic hub, followed by An Giang (25.1%) and Kien Giang (19.9%), ensuring a diverse representation.

**Table 1.** Sample characteristics.

Characteristic	Category	Frequency (n)	Percentage (%)
Geographic Location	Can Tho City	145	40.1
	An Giang Province	91	25.1
	Kien Giang Province	72	19.9
	Other Provinces	54	14.9
Industry Sector	Agriculture, Aquaculture, & Food Processing	128	35.4
	Services (e.g., tourism, logistics)	107	29.6
	Manufacturing (non-food)	72	19.9
	Other (e.g., construction, retail)	55	15.1
Firm Size (by employees)	Micro (< 10)	54	15.0
	Small (10–49)	200	55.2
	Medium (50–249)	108	29.8
Firm Age (years)	< 5 years	72	19.9
	5–10 years	128	35.3
	> 10 years	162	44.8
Respondent’s Position	Director/Owner	218	60.2
	Chief Accountant/Finance Manager	106	29.3
	Other Manager (e.g., Operations)	38	10.5

Source: Data Analysis Results

**Table 2.** Measurement model assessment: Reliability and convergent validity.

Construct	Item	Outer loadings	Cronbach’s alpha	Composite reliability	Average variance extracted	Proposed references
Stakeholder Pressure	SP1	0.809	0.841	0.893	0.676	Henriques & Sadorsky (1999) and Buysse & Verbeke (2003)
	SP2	0.820				
	SP3	0.830				
	SP4	0.831				
Leadership Commitment	LC1	0.924	0.868	0.910	0.718	Daily & Huang (2001) and Collier & Esteban (2007)
	LC2	0.816				
	LC3	0.824				
	LC4	0.819				
Accounting Department Competence	ADC1	0.831	0.808	0.873	0.633	Gray et al. (1996) and Schaltegger & Burritt (2000)
	ADC2	0.774				
	ADC3	0.766				
	ADC4	0.809				
Export Orientation	EO1	0.847	0.727	0.840	0.638	Cavusgil & Zou (1994)
	EO2	0.827				
	EO3	0.717				
SRA Implementation	SRAI1	0.851	0.845	0.896	0.683	Clarkson et al. (2008) and the GRI framework
	SRAI2	0.791				
	SRAI3	0.863				
	SRAI4	0.799				
Firm Performance	FP1	0.788	0.835	0.889	0.668	Powell (1995), building upon Dess & Robinson (1984)
	FP2	0.832				
	FP3	0.824				
	FP4	0.824				
Access to Finance	AF1	0.866	0.865	0.907	0.710	Beck et al. (2006) and the World Bank Enterprise Surveys
	AF2	0.873				
	AF3	0.756				
	AF4	0.869				

Source: Data Analysis Results

In terms of industry, the sample reflects the economic structure of the Mekong Delta, with a large proportion of firms from the Agriculture, Aquaculture, & Food Processing sector (35.4%), followed by Services (29.6%) and Manufacturing (19.9%). The majority of responding firms were small-sized enterprises (10–49 employees; 55.2%) and had been in operation for more than five years (80.1%), indicating a sample of established SMEs rather than new startups. Crucially, the respondents were primarily in key decision-making roles, with 60.2% being Directors or Owners and an additional 29.3% being Chief Accountants or Finance Managers. This ensures that the data were provided by individuals with deep knowledge of their firm’s strategic direction and financial operations, thus enhancing the validity of the findings.

### 4.2 Measurement model

The assessment of the reflective measurement model confirmed its quality. As shown in Table 2, all indicator loadings exceeded the recommended 0.70 threshold. The composite reliability (CR) values for all constructs ranged from 0.840 to 0.910, and Cronbach’s alpha (CA) values ranged from 0.727 to 0.868, both well above the 0.70 benchmark, thus confirming high internal consistency reliability ( Hair et al., 2019). Convergent validity was established, as the Average Variance Extracted (AVE) for all constructs exceeded the required minimum of 0.50.

Discriminant validity was confirmed using the Heterotrait-Monotrait (HTMT) ratio criterion. As shown in Table 3, all HTMT values were below the conservative threshold of 0.85 ( Henseler et al., 2015), indicating that each construct is empirically distinct.

**Table 3.** Discriminant validity assessment: Heterotrait-monotrait ratio (HTMT).

Construct	1	2	3	4	5	6	7
ADC							
AF	0.231						
EO	0.121	0.095					
FP	0.164	0.097	0.275				
LC	0.300	0.119	0.275	0.294			
SP	0.321	0.107	0.159	0.165	0.277		
SRAI	0.447	0.338	0.474	0.498	0.529	0.481	

Source: Data Analysis Results

### 4.3 Structural model

Prior to hypothesis testing, collinearity was assessed. All inner model Variance Inflation Factor (VIF) values were below the threshold of 5, ranging from 1.000 to 1.150, confirming that multicollinearity was not a concern ( Hair et al., 2019).

To test the hypotheses, a bootstrapping procedure with 5,000 resamples was performed. The results of the structural model assessment are presented in Table 4. The model explained a substantial portion of the variance in SRA Implementation (R ^2^ = 0.416), and a significant portion of the variance in Firm Performance (R ^2^ = 0.181) and Access to Finance (R ^2^ = 0.092).

**Table 4.** Structural model results: Hypothesis testing.

Hyp.	Path	Path Coeff. (β)	T-Value	P-Value	f ^2^	Decision
H1	SP - > SRAI	0.249	5.834	0.000	0.094	Supported
H2	LC - > SRAI	0.277	6,809	0.000	0.115	Supported
H3	ADC - > SRAI	0.213	5,180	0.000	0.069	Supported
H4	EO - > SRAI	0.279	6,930	0.000	0.126	Supported
H5	SRAI - > FP	0.425	10,454	0.000	0.221	Supported
H6	SRAI - > AF	0.303	5,867	0.000	0.101	Supported

*Note*: R ^2^(SRAI) = 0.416; R ^2^(FP) = 0.181; R ^2^(AF) = 0.092. Significance at p < 0.001.

Source: Data Analysis Results

**Figure 2.**
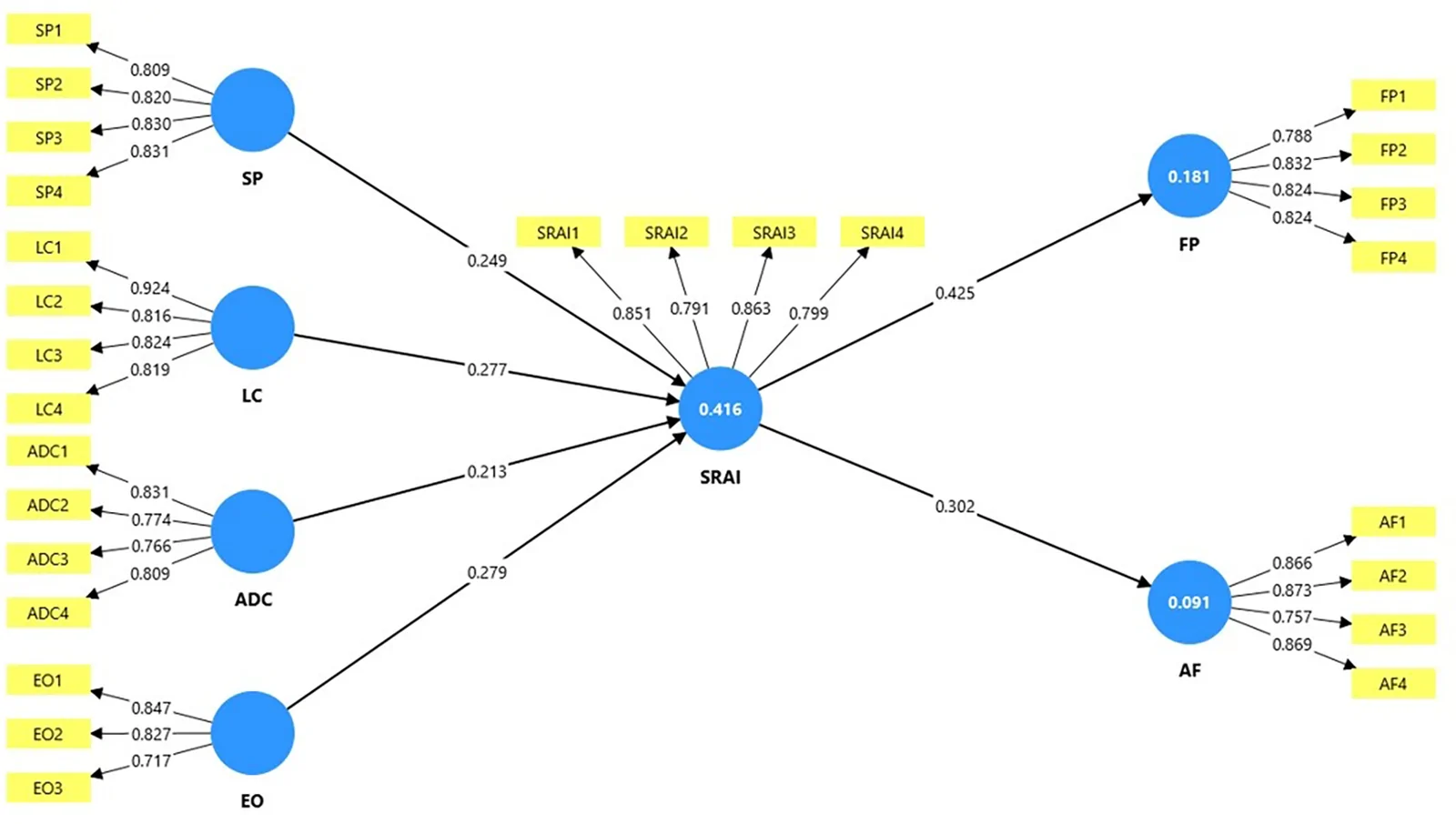
The antecedents and outcomes model of SRA implementation. Source: Data Analysis Results.

The testing hypothesis results support all six proposed relationships. The structural model and path coefficients are visually summarized in Figure 2. Specifically, Stakeholder Pressure (β = 0.249, t = 5.834, p < 0.001), Leadership Commitment (β = 0.277, t = 6.809, p < 0.001), Accounting Department Competence (β = 0.213, t = 5.180, p < 0.001), and Export Orientation (β = 0.279, t = 6.930, p < 0.001) all had a significant positive influence on SRA Implementation, thus supportingH1, H2, H3, and H4. In turn, SRA Implementation demonstrated a significant positive effect on both Firm Performance (β = 0.425, t = 10.454, p < 0.001) and Access to Finance (β = 0.303, t = 5.867, p < 0.001), providing strong support for H5 and H6.

## 5. Discussion

This section discusses the empirical findings, elaborates on their theoretical and practical implications, and outlines the study’s limitations, providing avenues for future research.

### 5.1 Discussion of findings

The primary objective of this study was to develop and test a comprehensive model of the antecedents and consequences of Social Responsibility Accounting (SRA) implementation within the specific context of Small and Medium-sized Enterprises (SMEs) in an emerging economy. The empirical results from the PLS-SEM analysis provide robust support for the proposed theoretical framework, with all six hypotheses being statistically significant.

First, regarding the antecedents of SRA implementation, the findings reveal that all four proposed drivers—stakeholder pressure, leadership commitment, accounting department competence, and export orientation—are significant positive predictors. Notably, Export Orientation (β = 0.279, t = 6.930) and Leadership Commitment (β = 0.277, t = 6.809) emerged as the most influential drivers. The prominence of export orientation strongly corroborates Institutional Theory, which suggests that firms conform to the norms and requirements of powerful external entities to gain legitimacy and resources ( DiMaggio & Powell, 1983). For SMEs in Vietnam, access to demanding international markets is often contingent on adherence to global standards for sustainability and transparency, making SRA a strategic necessity rather than a voluntary choice.

The strong influence of leadership commitment aligns perfectly with Upper Echelons Theory ( Hambrick & Mason, 1984), which is particularly salient in the SME context. Given the often centralized and hierarchical structure of SMEs, the owner-manager’s personal values and strategic vision are paramount in directing firm resources towards non-mandatory activities like SRA. This finding underscores that a proactive, top-down approach is a critical catalyst for embedding SRA into an SME’s operational fabric.

The significant effects of Stakeholder Pressure (β = 0.249, t = 5.834) and Accounting Department Competence (β = 0.213, t = 5.180) provide a more nuanced understanding. While stakeholder pressure acts as the external “push” for SRA adoption, accounting competence represents the internal “capability” required to implement it effectively. This dual finding supports an integrated view where external legitimacy-seeking pressures (Stakeholder Theory) must be matched with internal firm-specific capabilities (Resource-Based View) for strategic actions to be realized ( Schaltegger & Burritt 2010).

Second, concerning the outcomes, this study makes a significant contribution by empirically demonstrating the performance benefits of SRA. The path from SRA Implementation to Firm Performance (β = 0.425, t = 10.454) was the strongest in the entire model, with a medium effect size (f ^2^ = 0.221). This provides compelling evidence for the “doing well by doing good” argument, suggesting that systematic accounting for social and environmental impacts is not merely a compliance cost but a strategic investment that yields tangible performance returns, such as enhanced reputation and improved operational efficiency ( Porter & Kramer, 2006).

Furthermore, the significant positive path from SRA Implementation to Access to Finance (β = 0.303, t = 5.867) supports Signaling Theory. By implementing SRA, SMEs can transmit credible signals to financial institutions about their proactive risk management and long-term orientation, thereby reducing information asymmetry and perceived credit risk. This is a critical finding for SMEs, which traditionally face significant constraints in accessing external capital.

### 5.2 Theoretical implications

This study offers several key theoretical contributions.

First, it develops and validates an integrated, multi-theoretical model that explains both the drivers and outcomes of SRA. By combining Stakeholder Theory, Upper Echelons Theory, the Resource-Based View, Institutional Theory, and Signaling Theory, our research provides a more holistic and nuanced understanding of the SRA phenomenon than single-theory studies.

Second, by focusing on SMEs within an emerging economy (Vietnam), this study extends the generalizability of these established theories to a context that is critically under-researched. The findings confirm that while the theoretical mechanisms are robust, the relative importance of certain drivers (export orientation) is context-dependent.

Third, our model empirically establishes SRA implementation as a key strategic mediator. It acts as the mechanism through which external pressures and internal capabilities are converted into valuable firm-level outcomes, such as superior performance and enhanced access to finance.

### 5.3 Managerial and policy implications

The findings yield actionable insights for both SME managers and policymakers.

**For SME Managers:**

Strategic View of SRA: Leaders should reframe SRA from a compliance burden to a strategic tool for value creation. Our results show it directly links to improved performance and financial access.

Invest in Capabilities: The importance of accounting competence highlights the need for investing in training for financial personnel to handle non-financial data and reporting standards.

Leverage SRA for Market Access: For export-oriented SMEs, robust SRA practices can be a powerful differentiator and a key to entering or strengthening their position in global supply chains.

**For Policymakers:**

Promote and Support: Government agencies and business associations should develop targeted programs to raise awareness of SRA’s benefits and provide technical support for its implementation in SMEs.

Create Incentives: Policies could include preferential access to public contracts, tax incentives, or favorable loan conditions for SMEs that demonstrate transparent and high-quality SRA, thereby creating a virtuous cycle.

### 5.4 Limitations and future research

This study is not without limitations, which in turn suggests avenues for future research. First, the cross-sectional design allows us to establish associations but not definitive causality. Future longitudinal studies could track SMEs over time to better understand the causal dynamics between SRA implementation and performance. Second, the data were collected from a single city in Vietnam, which may limit the generalizability of the findings to other regions or countries. Comparative studies across different provinces in Vietnam or other Southeast Asian economies would be a valuable extension. Third, the use of self-reported, perceptual data for performance and other constructs could be subject to common method bias. Future research could seek to integrate objective data (financial statements, export ratios from customs data) to corroborate these findings. Finally, our model, while robust (R ^2^ = 0.416 for SRAI), does not capture all the variance, indicating that other factors may be at play. Future studies could incorporate other variables such as firm age, industry type, or the intensity of local competition to further enrich the model.

## Ethical approval

At the time of this study, Tay Do University did not have a formal Institutional Review Board (IRB) for non-medical social science research. Therefore, the authors strictly adhered to international ethical standards for research involving human participants, including the Declaration of Helsinki and the ESOMAR International Code on Market, Opinion and Social Research. The research design was meticulously crafted to ensure zero risk to participants. All respondents were SME managers who participated voluntarily and anonymously. No sensitive personal data or health-related information was collected. The primary ethical consideration was the protection of corporate data and respondent identity, which was achieved through full anonymization during the data processing stage. Participants were informed that they could withdraw from the survey at any point without any consequences.

## Data Availability

The dataset supporting the findings of this study is openly available in Zenodo at: https://zenodo.org/records/19720344 ( Nguyen et al., 2024). Drivers and Performance Outcomes of Social Responsibility Accounting: SMEs in the Mekong Delta, Vietnam. Data are available under the terms of the Creative Commons Attribution 4.0 International license (CC-BY 4.0).
