# Supplementary material for: Drivers and Performance Outcomes of Social Responsibility Accounting: An Integrated Model for SMEs in the Mekong Delta
Source: F1000Res. 2026 Jun 25;15:1017. [Version 1] doi: 10.12688/f1000research.179819.1 (PMC13562998; doi:10.12688/f1000research.179819.1)
Supplement: Peer review history

# Reviewer report: Reviewer response for version 1

**DOI:** 10.5256/f1000research.198370.r507617

**Published:** 2026-09-08

**Authors:** Junaid M Shaikh

**Affiliations:** 1 Universiti Teknologi Brunei, Bandar Seri Begawan, Brunei-Muara District, Brunei

This well-structured paper provides highly valuable insights into how social responsibility accounting drives performance in Mekong Delta SMEs. The integrated model is robust and contextually relevant. However, the study would benefit from a more detailed discussion on regional regulatory challenges. Overall, it offers a strong, significant contribution to sustainability literature.

Is the work clearly and accurately presented and does it cite the current literature?

Yes

If applicable, is the statistical analysis and its interpretation appropriate?

Yes

Are all the source data underlying the results available to ensure full reproducibility?

Yes

Is the study design appropriate and is the work technically sound?

Yes

Are the conclusions drawn adequately supported by the results?

Yes

Are sufficient details of methods and analysis provided to allow replication by others?

Yes

Reviewer Expertise:

ALL AREA  OF ACCOUNTING AND  FINANCE  INCLUDING TAX AND AUDITING  WITH RELATED ISSUES

I confirm that I have read this submission and believe that I have an appropriate level of expertise to confirm that it is of an acceptable scientific standard.

# Reviewer report: Reviewer response for version 1

**DOI:** 10.5256/f1000research.198370.r507609

**Published:** 2026-09-02

**Authors:** Galvarino Casanueva-Yáñez

**Affiliations:** 1 University of Las Americas, Santiago, Chile

**Report**

 Small and medium-sized enterprises in emerging economies often operate with limited resources, so the adoption of sustainability practices and accountability for social and environmental impacts are not automatic or homogeneous. In this scenario, the article on social responsibility accounting in the Mekong Delta proposes an integrated explanation of why it is implemented and the effects it produces once it ceases to be an intention and becomes an organizational practice.

 The work starts from a clear gap: while research on social responsibility accounting has advanced strongly in large corporations, knowledge about its background and consequences in SMEs is still insufficient. To fill that gap, the study is located in Vietnam, specifically in a region where the economic weight of SMEs is combined with considerable environmental vulnerability. This matters because companies not only face demands for legitimacy and compliance, but also experience the direct impact of their operational decisions on the environment, making changes towards responsible practices more urgent.

 From the theoretical approach, adoption is understood as a phenomenon that arises from the interaction between external forces, internal capabilities and strategic decisions. Stakeholder pressure is a relevant driver, as the company seeks credibility and continuity in contexts where customers, regulators, and communities demand evidence and transparency. In addition, export orientation is presented as a channel through which the requirements linked to responsible sourcing and standards from international markets are intensified. In this sense, social responsibility accounting works as an organizational response that allows external expectations to be translated into internal information and routines.

 However, environment alone does not explain behavior. The article underlines that SMEs also need capacities to consistently manage non-financial measurement and communication. Here, the competence of the accounting department represents a decisive internal resource, in line with the resource-based vision, since technical and organizational skill makes it possible to turn commitments into concrete processes. In the same way, leadership commitment becomes critical, because without strategic direction it is difficult to sustain change, allocate resources and maintain implementation over time.

 The empirical results support this logic. The implementation of social responsibility accounting is associated with better levels of business performance and greater access to financing. This suggests that accountability is not only a cost of compliance, but also a signal that improves the trust of financial actors while also driving internal improvements in control, management, and reputation. Overall, the study offers a view that connects adoption factors with concrete outcomes and shows that, for SMEs in the Mekong Delta, social responsibility accounting can operate as a strategic mechanism that transforms pressures and capabilities into organizational advantages.

 Strengths

- Relevant and little-explored approach: focuses social responsibility accounting on SMEs, where evidence is usually lower than in large companies.
- Context with high relevance: the Mekong Delta combines real environmental pressure and economic relevance, which makes the issue especially significant.
- Integrated theoretical model: combines theories (institutional, stakeholders, resources, leadership and signaling) that allows explaining antecedents and consequences from different perspectives.
- Solid methodological design for the objective: sequential mixed method (interviews to build the instrument and then a large survey) and use of PLS-SEM to test the relationships between variables.
- Results with managerial utility: not only does it explain adoption, it also demonstrates impacts on performance and access to financing, which is valuable for decision-making.

**Article suitable for indexing by:**

 Contribute to a real gap: SRC in SMEs in an emerging context with environmental vulnerability (Mekong Delta).

 Integrated and well-founded theoretical model (several theories to explain the background and consequences).

 Quantitative evidence with a structural approach: PLS-SEM is used with a relatively large sample (362 SMEs).

 Results with strong practical implications: connect CRS with performance and access to finance, which is often of interest in "management/finance/sustainability".

 Undoubtedly, the study, if continued, has room for improvement.

Is the work clearly and accurately presented and does it cite the current literature?

Yes

If applicable, is the statistical analysis and its interpretation appropriate?

Yes

Are all the source data underlying the results available to ensure full reproducibility?

Yes

Is the study design appropriate and is the work technically sound?

Yes

Are the conclusions drawn adequately supported by the results?

Yes

Are sufficient details of methods and analysis provided to allow replication by others?

Yes

Reviewer Expertise:

SMEs, Entrepreneurship

I confirm that I have read this submission and believe that I have an appropriate level of expertise to confirm that it is of an acceptable scientific standard.
